# Exercise Performance and Thermoregulatory Responses of Elite Athletes Exercising in the Heat: Outcomes of the Thermo Tokyo Study

**DOI:** 10.1007/s40279-021-01530-w

**Published:** 2021-08-15

**Authors:** Johannus Q. de Korte, Coen C. W. G. Bongers, Maria T. E. Hopman, Thijs M. H. Eijsvogels

**Affiliations:** Department of Physiology (392), Radboud University Medical Centre, Radboud Institute for Health Sciences, P.O. Box 9101, 6500 HB Nijmegen, The Netherlands

## Abstract

**Objective:**

We examined the impact of simulated Tokyo 2020 environmental condition on exercise performance, thermoregulatory responses and thermal perception among Dutch elite athletes.

**Methods:**

105 elite athletes from different sport disciplines performed two exercise tests in simulated control (15.9 ± 1.2 °C, relative humidity (RH) 55 ± 6%) and Tokyo (31.6 ± 1.0 °C, RH 74 ± 5%) environmental conditions. Exercise tests consisted of a 20-min warm-up (70% HR_max_), followed by an incremental phase until volitional exhaustion (5% workload increase every 3 min). Gastrointestinal temperature (*T*_gi_), heart rate, exercise performance and thermal perception were measured.

**Results:**

Time to exhaustion was 16 ± 8 min shorter in the Tokyo versus the control condition (− 26 ± 11%, whereas peak power output decreased with 0.5 ± 0.3 W/kg (16 ± 7%). Greater exercise-induced increases in *T*_gi_ (1.8 ± 0.6 °C vs. 1.5 ± 0.5 °C, *p* < 0.001) and higher peak *T*_gi_ (38.9 ± 0.6 °C vs. 38.7 ± 0.4 °C, *p* < 0.001) were found in the Tokyo versus control condition. Large interindividual variations in exercise-induced increase in *T*_gi_ (range 0.7–3.5 °C) and peak *T*_gi_ (range 37.6–40.4 °C) were found in the Tokyo condition, with greater *T*_gi_ responses in endurance versus mixed- and skill-trained athletes. Peak thermal sensation and thermal comfort scores deteriorated in the Tokyo condition, with aggravated responses for power versus endurance- and mixed-trained athletes.

**Conclusion:**

Large performance losses and *T*_gi_ increases were found among elite athletes exercising in simulated Tokyo conditions, with a substantial interindividual variation and significantly different responses across sport disciplines. These findings highlight the importance of an individual approach to optimally prepare athletes for safe and maximal exercise performance during the Tokyo Olympics.

**Supplementary Information:**

The online version contains supplementary material available at 10.1007/s40279-021-01530-w.

## Key Points

**Table:** 

Tokyo’s climate has a major impact on the exercise capacity of non-acclimatized elite athletes, independent of sports discipline
A large interindividual variability was found for exercise capacity and thermoregulatory responses, whereas no significant association was found between peak core temperature and performance outcomes
Findings from this study emphasize that it is impossible to offer a ‘one-size-fits-all’ heat mitigation strategy to elite athletes, and underline the importance of determining the individual’s needs for heat acclimatization, cooling interventions and a hydration plan

## Introduction

The 2020 Summer Olympics will be held amidst Tokyo’s summer, characterised by hot [ambient air temperature (*T*_*a*mbient_) > 30 °C] and humid [relative humidity (RH) ± 70%] environmental conditions, presumably resulting in the most challenging environmental conditions ever observed during the Olympic (Summer) Games [1, 2]. The combination of environmental heat stress and exercise-induced heat production is likely to exceed the body’s heat-dissipating capabilities [3, 4], resulting in profound core temperature elevations [4–6]. Exercise-induced core temperature elevations may cause substantial reductions in exercise performance [7, 8], and place athletes at risk of developing heat-related illnesses such as heat exhaustion and heat stroke [4, 9, 10].

Previous field studies showed considerable heterogeneity in exercise-induced thermoregulatory responses across athletes [6, 11, 12], but smaller within-participant variations [13]. These findings suggest that thermoregulatory responses during exercise in the heat might be individually determined. Indeed, a pilot study among Olympic sailors reported that some athletes were particularly prone to large performance losses but others to a high end-exercise core temperature [14]. Heat acclimatization and cooling strategies are effective countermeasures to optimize exercise performance in the heat and to minimize the risk of heat-related illnesses [15–17]. However, recommendations are often based on a one-size-fits-all approach, while the needs of an individual athlete for heat mitigation strategies may differ substantially based on their physiological responses to heat stress [18]. Identification of elite athletes with a low heat tolerance is, therefore, of utmost importance to allow timely interventions (i.e., heat acclimatization and cooling strategies) prior to Tokyo 2020 [18].

The aim of the Thermo Tokyo study was to examine the impact of the Tokyo environmental conditions on exercise performance and thermoregulatory responses in non-acclimatized elite athletes. We were specifically interested in the quantification of interindividual variations and differences in physiological and perceptual responses across sport disciplines (skill, power, mixed and endurance), as anthropometric (i.e. body fat and surface to mass ratio) and physiological (i.e. *V*O_2max_, peak power output) parameters are known to influence individual responses to heat stress [19] and may differ across sport disciplines [20]. Although observational field studies have a high external validity, such a study design hinders the assessment of heat stress-induced performance loss and prevents a direct comparison across different sport disciplines and different ambient conditions due to a low internal validity. Therefore, we performed a controlled exercise protocol in simulated Tokyo 2020 environmental conditions in the climate chamber of the Dutch Olympic Training Centre.

## Methods

### Participants

Dutch elite athletes were recruited via TeamNL (the Olympic division of all Dutch sports federations) infrastructures (i.e. via national sports federations, coaches, embedded scientists). Elite athletes ≥ 16 years old and practicing an outdoor sport discipline on an international level were eligible to participate in our study. Exclusion criteria were based on the use of the ingestible temperature capsule: (i) a bodyweight < 36.5 kg, (ii) an implanted electro-medical device, (iii) a history of obstructive/inflammatory bowel disease or surgery, or (iv) a scheduled MRI scan within 5 days of the experiment. Based on the relative isometric and isotonic components of exercise training, participants were classified as endurance, mixed, power or skill athletes (Table 1) [21]. The study was carried out in accordance with the Declaration of Helsinki and was approved by the Medical Ethical Committee of the Radboud University Medical Centre (#2018-4640). All participants gave their written informed consent prior to the testing procedures.

**Table 1:** Athlete characteristics of the whole cohort as well as specific sport disciplines

	Endurance-trained athletes (*N* = 27)	Mixed-trained athletes (*N* = 31)	Power-trained athletes (*N* = 12)	Skill-trained athletes (*N* = 35)	*p* value	All athletes (*N* = 105)
Age, years	25 ± 6	27 ± 4^c^	23 ± 3^b^	25 ± 5	0.029*	26 ± 5
Sex, *n* (% male)	12 (44)	22 (71)^d^	7 (58)	11 (31)^b^	0.011*	52 (50)
Height, cm	176 ± 10^b^	187 ± 13^a,c,d^	176 ± 8^b^	177 ± 10^b^	< 0.001*	180 ± 12
Weight, kg	65.5 ± 9.4^b,c,d^	81.3 ± 13.2^a^	78.6 ± 10.0^a^	78.9 ± 14.7^a^	< 0.001*	76.1 ± 13.9
BMI, kg/m^2^	20.9 ± 1.4^b,c,d^	23.1 ± 1.5^a,c,d^	25.4 ± 1.3^a,b^	24.9 ± 3.0^a,b^	< 0.001*	23.4 ± 2.7
BSA, m^2^	1.81 ± 0.18^b,d^	2.07 ± 0.24^a^	1.95 ± 0.17	1.96 ± 0.22^a^	< 0.001*	1.95 ± 0.23
Sport disciplines	Mountain biking *n* = 5, open water swimming *n* = 2, road cycling *n* = 7, triathlon *n* = 13	3 x 3 basketball *n* = 7, beach volleyball *n* = 8, field hockey *n* = 15, soccer *n* = 1	BMX *n* = 12	Baseball *n* = 11 Sailing *n* = 3 Skateboarding *n* = 1 Softball *n* = 20		

Data are presented as mean ± SD or *n* (%)

*BMI* body mass index, *BSA* body surface area

*p < 0.05. Significantly different from (a) endurance-trained athletes, (b) mixed-trained athletes, (c) power-trained athletes, (d) skill-trained athletes

### Design

The current study is part of the Thermo Tokyo research project, the rationale and design of which have been described previously [22]. In short, athletes were invited to complete two personalised incremental exercise tests on a cycling ergometer (Lode ergometer, Lode B.V., Groningen, the Netherlands, or Tacx Neo Smart T2800, Tacx B.V., Wassenaar, the Netherlands) in a climate chamber until volitional exhaustion. Exercise tests were conducted in control (*T*_ambient_ 15.9 ± 1.2 °C, RH 55 ± 6%, ambient vapour pressure 0.99 kPa, absolute humidity 0.0075 kg/m^3^) and Tokyo (*T*_ambient_ 31.6 °C ± 1.0, RH 74 ± 5%, ambient vapour pressure 3.45 kPa, absolute humidity 0.0245 kg/m^3^) environmental conditions. The first exercise test was always conducted in the control condition and an ambient temperature of 15 °C was used to simulate the average ambient temperature during the summer months in the Netherlands. The personalised exercise protocol (i.e. changes in workload over time) obtained during the control condition was subsequently applied to the second exercise test in simulated Tokyo conditions. Through this, the difference between the Tokyo and control condition demonstrates the impact of the Tokyo environment on exercise performance and thermoregulatory responses compared to Dutch reference standards in non-acclimatized athletes. Study visits were separated by > 48 h, and in preparation for each visit, participants were instructed to refrain from strenuous exercise (24 h) and consumption of alcohol or caffeine (12 h). Furthermore, all participants were instructed to eat the same diet from the moment of awakening onwards and to wear the same clothes for both exercise tests, consume their last meal ≥ 3 h preceding the measurements, and consume 500 mL of water ∼ 2 h before arriving at the laboratory. Study visits commenced at the same time of day to avoid any circadian rhythm effects [23]. Participants ingested a gastrointestinal temperature capsule (myTemp, Nijmegen, the Netherlands) ~ 3 h prior to both study visits and were not allowed to drink during the exercise protocol to avoid any interaction with fluid intake [24].

### Personalised Exercise Test Protocol

After the participant entered the climate chamber, the bike ergometer was fitted to the participant by adjusting the height, inclination and position of the saddle, and the height and inclination of the handlebar. If a Tacx device was used, the participant’s bike was installed before entering the climate chamber. The ergometer type and settings were the same for both tests. Then, while seated on the ergometer, the 5-min seated rest period was applied and baseline values were obtained during the last minute. Thereafter, athletes started the warm-up phase by exercising at 100 W and were instructed to maintain a pedalling speed of 80–100 revolutions per minute throughout the whole protocol. On the 3-min mark, the initial workload (in W) was gradually adjusted to reach 70% of the individual athlete's maximal heart rate (HR), which was obtained from training data or a previously performed maximal exercise test. Workload adjustments (in W) were performed on the minute marks and repeated (if necessary) until a stable target HR (i.e. 70% HRmax) was reached. Then, the workload was kept equal for the remaining minutes of the 20-min warm-up phase. At the 20-min mark, the incremental phase started and the workload (in W) was increased every 3 min by 5% of the workload corresponding to 70% HRmax. The incremental phase lasted until volitional exhaustion was reached. After exercise cessation, participants had a 3-min active cool-down at a self-selected wattage, followed by a 10-min seated rest. A protocol overview is presented in Supplementary Fig. 1 (OSM) and an example of a personalised protocol is presented in Supplementary Table 1 (OSM).

### Measurements

#### Exercise Performance

Time to exhaustion (TTE) was measured from the start of the warm-up until volitional exhaustion and was expressed in minutes rounded to the nearest integer. Peak power output (PPO) was expressed as an absolute (W) and normalized value (W/kg), and was calculated using the following formula:

$$\begin{gathered} {\text{PPO }}\left( {\text{W}} \right) = {\text{workload in the highest completed step }}\left( {\text{W}} \right) \hfill \\ + \left( {\left( {{\text{time in the higest incomplete step }}\left( {\text{s}} \right) /{\text{step duration}}} \right) \times {\text{additional workload in the highest incomplete step}} \left( {\text{W}} \right)} \right) . \hfill \\ \end{gathered}$$

Changes in TTE (min) and PPO (W and W/kg) in the Tokyo condition relative to the control condition were calculated using the following formula:

$${\text{Change in exercise performance}} = \left( {{\text{Tokyo}} {-}{\text{ control}}} \right) / {\text{control}} \times 100\% .$$

#### Gastrointestinal Temperature (*T*_gi_)

We used a validated ingestible telemetric temperature capsule system (myTemp, Nijmegen, the Netherlands) [25, 26] to continuously measure *T*_gi_ (in °C) at predefined 10-s intervals. The calibration of the myTemp ingestible temperature capsules was performed by the manufacturer (myTemp, Nijmegen, the Netherlands) as additional calibration prior to use has been demonstrated to be unnecessary [25].

#### Skin Temperature (*T*_sk_)

Wireless temperature recorders (iButton DS1922L, Dallas Semiconductor Corp, USA) were attached to the skin at four distinct locations (i.e. neck, left hand, right shoulder, right shin) [27] using sweat proof Tegaderm Film (Tegaderm, Neuss, Germany) to determine *T*_sk_. Resolution was set at 0.0625 °C and data were continuously collected at 20-s intervals. Weighted averages were calculated according to international standard operations (ISO-9886) [27].

#### Heart Rate (HR)

HR was measured using a Polar system (Polar V800, Polar Electro Oy, Kempele, Finland) using 1-s intervals.

#### Whole Body Sweat Rate (WBSR)

Participants’ bodyweight was measured to the nearest 100 g using an electronic weighting scale (Seca robusta 813 scale, Hamburg, Germany) at baseline and directly after finishing the experimental protocol to determine WBSR. Bodyweight measurements were performed in shorts and underwear and the assessment of WBSR did not contain any fluid intake or urine excretion. Dehydration was defined as a bodyweight loss of > 2% [28].

#### Subjective Measures

Thermal comfort, thermal sensation and rating of perceived exertion (RPE) were ranked on a 4-point [29], 7-point [29] and 15-point scale [30], respectively. Thermal comfort ranged from 1 (comfortable) to 4 (very uncomfortable), Thermal sensation from − 3 (very cold) to 3 (very hot), and RPE from 6 (very very light) to 20 (maximal exertion). Subjective parameters were scored at baseline, every 5 min during the warm-up phase, every 3 min during the incremental phase and every 5 min during the recovery phase.

#### Environmental Conditions

*T*_ambient_ and RH were measured using a portable climate-monitoring device (Davis Instruments Inc., Hayward, CA, USA) positioned at table height in the centre of the climate chamber, and the ambient vapour pressure and absolute humidity were calculated accordingly.

### Statistical Analysis

Minute averages of *T*_gi_, *T*_sk_ and HR were calculated using a customized MATLAB and Statistics Toolbox (2012b, The MathWorks, Inc., Natick, MA, USA) software package. All parameters were visually inspected for normality. Continuous variables were normally distributed and presented as mean ± SD, whereas categorical variables were presented as median [interquartile range (IQR)] or as proportions. Paired-samples *t* tests were used to compare exercise characteristics and thermoregulatory responses between the control and Tokyo conditions. The Wilcoxon Signed ranks-test was used to compare subjective outcome measures between the control and Tokyo conditions. A one-way ANOVA with Bonferroni post hoc testing was used to compare exercise characteristics and thermoregulatory responses across sport disciplines. Kruskal–Wallis tests with the Dunn-Bonferroni post hoc method were used to compare subjective outcome measures across sport disciplines. Pearson’s correlation analysis was used to assess bi-variate associations. Statistical analyses were performed with SPSS Statistics 25 (IBM Corp, Armonk, NY, USA). Data were considered to be significant if *p* < 0.05.

## Results

### Participants

A total of 106 elite athletes from 11 different sport types participated in this study. One participant dropped out due to an ankle injury that was incurred during training and was excluded from further analyses. Participant characteristics of the analytical cohort (*n* = 105) and detailed information about classification of sport disciplines are presented Table 1. Due to technical difficulties, we missed *T*_gi_ observations in seven athletes (*n* = 3 in the control and *n* = 4 in the Tokyo condition). The exercise tests were conducted between 20 November 2018 and 30 April 2019 and between 21 October 2019 to 23 January 2020. In addition, two athletes were tested in June 2019 as they were not available earlier due to their intense training schedules. None of the participating athletes conducted a dedicated heat acclimatization program prior to participation and only four athletes reported having some heat exposure but were not acclimatized. The average time between study visits was 8 ± 6 days, with a minimum of 48 h.

### Exercise Performance

**Fig. 1:**
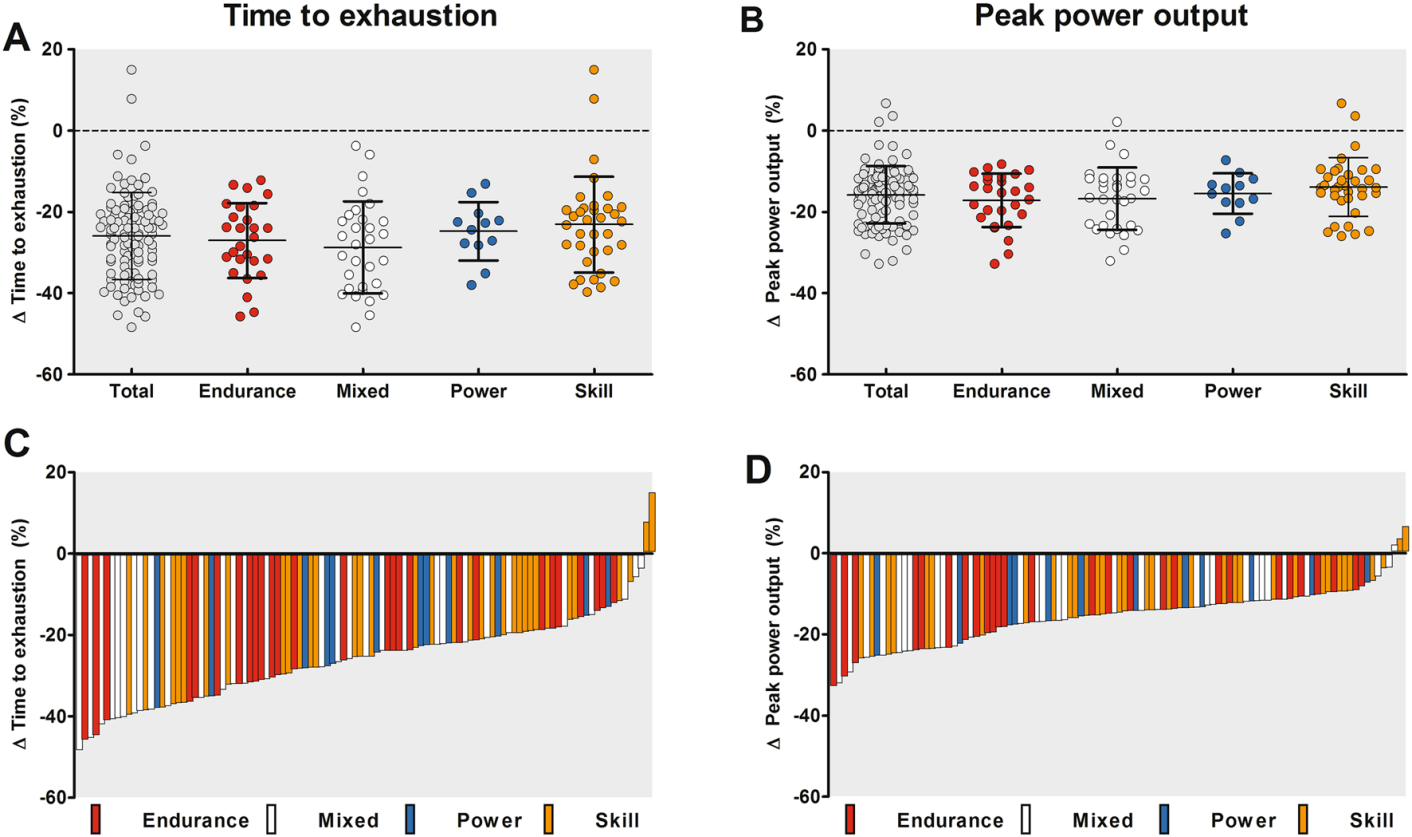
Group data (panels **A** + **B**) and individual data (panels **C** + **D**) of time to exhaustion (TTE) (panels **A** + **C**) and peak power output (PPO) (panels **B** + **D**) in the Tokyo condition relative to the control condition. Data in the upper panels are presented as mean ± SD. The relative changes in exercise performance were not different across sport disciplines. Each bar of panels **C** and **D** represent data from an individual athlete, highlighting the large interindividual variability in changes in exercise performance during exercise in the heat

TTE was 16 ± 8 min shorter in the Tokyo compared to the control condition (Table 2, *p* < 0.001), corresponding to a 26 ± 11% reduction in exercise performance. Accordingly, PPO declined with 0.5 ± 0.3 W/kg in the Tokyo versus control condition (Table 2, *p* < 0.001), corresponding to a 16 ± 7% reduction in exercise performance. The reductions in exercise performance were not different across sport disciplines (Fig. 1A, B, Table 3). Nevertheless, large variations in the magnitude of performance reductions between individual athletes were observed (Fig. 1C, D).

**Table 2:** Comparison of exercise characteristics and thermoregulatory responses between the control condition and the Tokyo condition

	Control condition	Tokyo condition	*p* value
Exercise characteristics			
Time to exhaustion (min)	60 ± 14	44 ± 10	< 0.001*
Peak power output (W)	230 ± 63	193 ± 54	< 0.001*
Peak power output (W/kg)	3.1 ± 1.0	2.6 ± 0.8	< 0.001*
Resting HR (bpm)	74 ± 12	82 ± 14	< 0.001*
Exercise-induced increase in HR (bpm)	105 ± 14	101 ± 15	0.005*
Peak HR (bpm)	179 ± 12	182 ± 11	< 0.001*
WBSR (L/h)	0.8 ± 0.3	1.4 ± 0.6	< 0.001*
Dehydration (%)	1.1 ± 0.4	1.3 ± 0.5	< 0.001*
Thermoregulatory responses			
Resting *T*_gi_ (°C)	37.1 ± 0.4	37.1 ± 0.4	0.23
Exercise-induced increase in *T*_gi_ (°C)	1.5 ± 0.5	1.8 ± 0.6	< 0.001*
Exercise-induced increase rate in *T*_gi_ (°C/h)	1.6 ± 0.5	2.5 ± 0.8	< 0.001*
Peak *T*_gi_ (°C)	38.7 ± 0.4	38.9 ± 0.6	< 0.001*
Resting *T*_sk_ (°C)	30.5 ± 0.7	33.6 ± 0.7	< 0.001*
Exercise-induced increase in *T*_sk_ (°C)	1.8 ± 0.9	3.1 ± 0.9	< 0.001*
Peak *T*_sk_ (°C)	32.3 ± 1.1	36.7 ± 0.6	< 0.001*
Subjective outcomes			
Resting thermal sensation (au)	− 2 (− 3 to 1)	1 (0–3)	< 0.001*
Peak thermal sensation (au)	3 (0–3)	3 (2–3)	< 0.001*
Resting thermal comfort (au)	2 (1–4)	1 (1–3)	< 0.001*
Peak thermal comfort (au)	4 (1–4)	4 (2–4)	< 0.001*
Resting RPE (au)	6 (6–10)	6 (6–10)	0.47
Peak RPE (au)	20 (14–20)	20 (12–20)	0.11

Data are presented as mean ± SD or median (interquartile range)

*au* arbitrary units, *bpm* beats per minute, *HR* heart rate, *RPE* rating of perceived exertion, *T*_*gi*_ gastrointestinal temperature, *T*_*sk*_ skin temperature, *W* watt, *WBSR* whole body sweat rate

*p < 0.05

**Table 3:** Comparison of exercise characteristics, thermoregulatory responses and subjective outcomes across sport disciplines

	Endurance-trained athletes (*N* = 27)	Mixed-trained athletes (*N* = 31)	Power-trained athletes (*N* = 12)	Skill-trained athletes (*N* = 35)	*p* value
Exercise characteristics					
Time to exhaustion (min)					
Control (min)	64 ± 13	57 ± 14	59 ± 6	60 ± 16	0.19
Tokyo (min)	47 ± 9	40 ± 10	44 ± 5	45 ± 11	0.05
Δ (min)	18 ± 9	17 ± 9	15 ± 5	14 ± 9	0.39
Δ time to exhaustion (%)	27 ± 9	29 ± 11	25 ± 7	23 ± 12	0.17
Peak power output (W)					
Control (W)	287 ± 50^b,c,d^	243 ± 50^a,d^	239 ± 51^a,d^	171 ± 31^a,b,c^	< 0.001*
Tokyo (W)	238 ± 46^b,d^	202 ± 44^a,d^	202 ± 44^d^	148 ± 30^a,b,c^	< 0.001*
Δ (W)	49 ± 19^d^	41 ± 21^d^	37 ± 15	24 ± 12^a,b^	< 0.001*
Δ peak power output (%)	17 ± 7	17 ± 8	16 ± 5	14 ± 7	0.24
Normalized peak power output (W/kg)					
Control (W/kg)	4.4 ± 0.5^b,c,d^	3.0 ± 0.4^a,d^	3.0 ± 0.4^a,d^	2.2 ± 0.5^a,b,c^	< 0.001*
Tokyo (W/kg)	3.7 ± 0.5^b,c,d^	2.5 ± 0.3^a,d^	2.6 ± 0.3^a,d^	1.9 ± 0.4^a,b,c^	< 0.001*
Δ (W/kg)	0.8 ± 0.3^b,c,d^	0.5 ± 0.3^a,d^	0.5 ± 0.2^a^	0.3 ± 0.2^a,b^	< 0.001*
Δ normalized peak power output (%)	17 ± 7	17 ± 8	16 ± 5	14 ± 7	0.24
Resting HR (bpm)					
Control (bpm)	72 ± 15	71 ± 9^c^	82 ± 11^b^	77 ± 11	0.002*
Tokyo (bpm)	75 ± 14^c,d^	78 ± 10^c^	95 ± 16^a,b^	85 ± 13^a^	< 0.001*
Δ (bpm)	3 ± 17	7 ± 11	12 ± 12	9 ± 9	0.14
Exercise-induced increase in HR (bpm)					
Control (bpm)	112 ± 15^d^	103 ± 11	106 ± 11	100 ± 13^a^	0.007*
Tokyo (bpm)	111 ± 12^b,c,d^	100 ± 13^a^	97 ± 18^a^	95 ± 14^a^	< 0.001*
Δ (bpm)	1 ± 18	3 ± 13	9 ± 14	5 ± 10	0.33
Peak HR (bpm)					
Control (bpm)	183 ± 10^b^	174 ± 9^a,c^	188 ± 6^b,d^	177 ± 13^c^	< 0.001*
Tokyo (bpm)	186 ± 9^b^	178 ± 12^a,c^	192 ± 9^b,d^	180 ± 11^c^	0.001*
Δ (bpm)	3 ± 5	4 ± 8	4 ± 6	4 ± 8	0.85
Thermoregulatory responses					
Resting *T*_gi_ (°C)					
Control (°C)	37.0 ± 0.4^c^	37.0 ± 0.3^c^	37.4 ± 0.3^a,b^	37.2 ± 0.3	0.006*
Tokyo (°C)	37.1 ± 0.3	37.0 ± 0.5	37.3 ± 0.3	37.2 ± 0.5	0.13
Δ (°C)	0.0 ± 0.3	0.1 ± 0.4	0.1 ± 0.2	0.0 ± 0.5	0.62
Exercise-induced increase in *T*_gi_ (°C)					
Control (°C)	1.7 ± 0.5^d^	1.6 ± 0.5	1.5 ± 0.3	1.4 ± 0.4^a^	0.048*
Tokyo (°C)	2.3 ± 0.6^b,d^	1.7 ± 0.6^a^	1.8 ± 0.6	1.6 ± 0.5^a^	< 0.001*
Δ (°C)	0.6 ± 0.4^d^	0.2 ± 0.5	0.3 ± 0.6	0.2 ± 0.6^a^	0.020*
Exercise-induced increase rate in *T*_gi_ (°C/h)					
Control (°C/h)	1.7 ± 0.6	1.7 ± 0.5	1.5 ± 0.3	1.5 ± 0.4	0.23
Tokyo (°C/h)	3.0 ± 0.9^d^	2.6 ± 0.6	2.5 ± 0.6	2.1 ± 0.5^a^	< 0.001*
Δ (°C/h)	1.4 ± 0.6^b,d^	0.9 ± 0.6^a^	0.9 ± 0.6	0.7 ± 0.6^a^	< 0.001*
Peak exercise *T*_gi_ (°C)					
Control (°C)	38.8 ± 0.4	38.6 ± 0.5	38.9 ± 0.3	38.6 ± 0.4	0.10
Tokyo (°C)	39.4 ± 0.5^b,d^	38.6 ± 0.5^a^	39.1 ± 0.5	38.8 ± 0.5^a^	< 0.001*
Δ (°C)	0.6 ± 0.4^b,d^	0.1 ± 0.4^a^	0.2 ± 0.4	0.2 ± 0.4^a^	< 0.001*
Resting *T*_sk_ (°C)					
Control (°C)	30.5 ± 0.7	30.4 ± 0.8	30.3 ± 0.6	30.6 ± 0.7	0.6
Tokyo (°C)	33.7 ± 0.6	33.8 ± 0.7	33.8 ± 0.6	33.3 ± 0.8	0.038*
Δ (°C)	3.1 ± 0.8	3.4 ± 0.9^d^	3.5 ± 0.7^d^	2.7 ± 0.7^b,c^	0.004*
Exercise-induced increase in *T*_sk_ (°C)					
Control (°C)	2.4 ± 0.8^b,d^	1.6 ± 0.7^a^	1.7 ± 1.3	1.4 ± 0.8^a^	< 0.001*
Tokyo (°C)	3.6 ± 0.7^b^	2.8 ± 0.7^a^	3.0 ± 0.9	3.1 ± 0.9	0.004*
Δ (°C)	1.1 ± 0.8	1.2 ± 1.0	1.2 ± 1.0	1.8 ± 1.0	0.046*
Peak exercise *T*_sk_ (°C)					
Control (°C)	33.0 ± 1.0^b,d^	32.0 ± 1.0^a^	32.1 ± 1.5	32.0 ± 0.9^a^	0.001*
Tokyo (°C)	37.3 ± 0.6^b,d^	36.6 ± 0.5^a^	36.8 ± 0.8	36.4 ± 0.5^a^	< 0.001*
Δ (°C)	4.3 ± 1.0	4.5 ± 1.0	4.7 ± 1.0	4.5 ± 0.9	0.62
Whole body sweat rate (WBSR)					
WBSR (L/h)					
Control (L/h)	0.9 ± 0.3^d^	0.9 ± 0.4^d^	0.8 ± 0.3	0.7 ± 0.2^a,b^	0.001*
Tokyo (L/h)	1.6 ± 0.6^c,d^	1.7 ± 0.6^c,d^	1.1 ± 0.3^a,b^	1.0 ± 0.3^a,b^	< 0.001*
Δ (L/h)	0.6 ± 0.3^d^	0.8 ± 0.4^c,d^	0.3 ± 0.2^b^	0.3 ± 0.3^a,b^	< 0.001*
Dehydration (%)					
Control (%)	1.4 ± 0.3^b,c,d^	1.0 ± 0.4^a^	1.0 ± 0.3^a^	0.8 ± 0.3 ^a^	< 0.001*
Tokyo (%)	1.8 ± 0.5^b,c,d^	1.3 ± 0.3^a,d^	1.0 ± 0.3^a^	0.9 ± 0.3^a,b^	< 0.001*
Δ (%)	0.3 ± 0.4^d^	0.3 ± 0.3^d^	0.1 ± 0.3	0.1 ± 0.3^a.b^	< 0.001*
Subjective measures					
Resting thermal sensation (au)					
Control (au)	− 2 (− 3 to 0)	− 2 (− 3 to 0)	− 2 (− 3 to 1)	− 1 (− 3 to 0)	0.14
Tokyo (au)	1 (0–3)	1 (0–2)	2 (0–3)	1 (0–2)	0.80
Peak thermal sensation (au)					
Control (au)	3 (0–3)	3 (1–3)	3 (2–3)	3 (1–3)	0.20
Tokyo (au)	3 (3–3)	3 (2–3)	3 (3–3)	3 (2–3)	0.46
Resting thermal comfort (au)					
Control (au)	2 (1–3)	2 (1–3)	2 (1–4)	2 (1–3)	0.25
Tokyo (au)	1 (1–3)^b,d^	1 (1–2)^a,c^	2 (1–3)^b,d^	1 (1–3)^a,c^	0.046*
Peak thermal comfort (au)					
Control (au)	4 (1–4)	4 (1–4)	4 (2–4)	3 (2–4)	0.326
Tokyo (au)	4 (3–4)^c^	4 (3–4)^c^	4 (4–4)^a,b^	4 (2–4)	0.031*
Resting RPE (au)					
Control (au)	6 (6–7)	6 (6–10)	6 (6–6)	6 (6–10)	0.22
Tokyo (au)	6 (6–8)	6 (6–8)	6 (6–6)	6 (6–10)	0.20
Peak RPE (au)					
Control (au)	20 (16–20)	20 (18–20)	20 (18–20)	20 (14–20)	0.29
Tokyo (au)	20 (12–20)	20 (17–20)	20 (18–20)	20 (15–20)	0.28

Data are presented as mean ± SD, *n* (%), or median (interquartile range)

*Δ* delta control versus Tokyo condition, *au* arbitrary units, *bpm* beats per minute, *HR* heart rate, *RPE* rating of perceived exertion, *T*_*gi*_ gastrointestinal temperature, *T*_*sk*_ skin temperature, *W* watt, *WBSR* whole body sweat rate

*p < 0.05. Significantly different from (a) endurance-trained athletes, (b) mixed-trained athletes, (c) power-trained athletes, (d) skill-trained athletes

### Thermoregulatory Responses

**Fig. 2:**
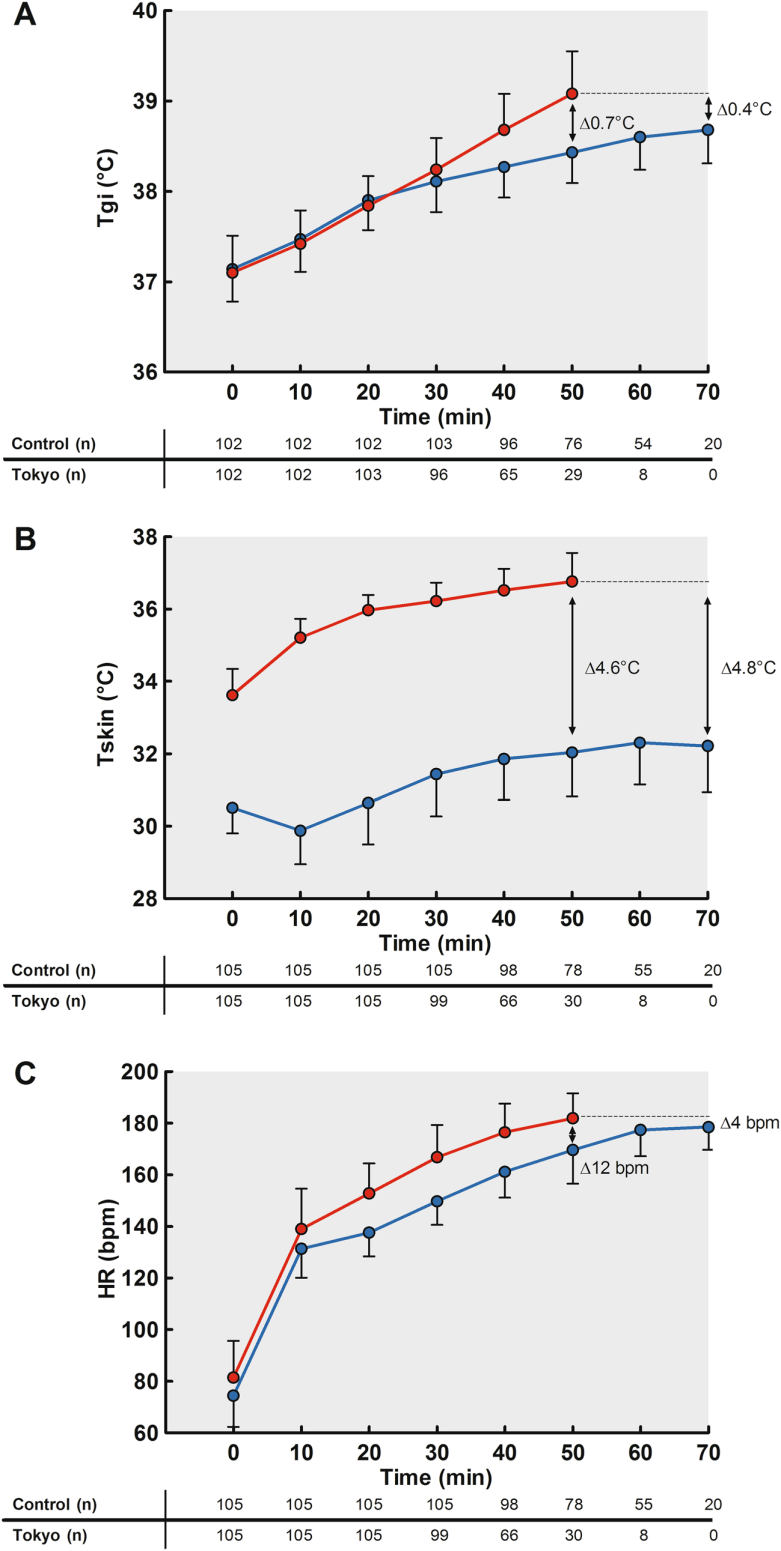
**A** Exercise-induced increases in gastrointestinal temperature (*T*_gi_), **B** skin temperature (*T*_sk_) and **C** heart rate (HR) during the control (blue lines) and Tokyo (red lines) conditions. **A** An increase in *T*_gi_ was observed in both conditions, with greater values in the Tokyo versus the control condition. **B*** T*_sk_ increased over time with greater values in the Tokyo versus the control condition. **C** HR values increased over time in both conditions, with higher values in the Tokyo versus the control condition. Data are presented as mean ± SD for all time points with a sample size > 10% of our cohort

**Fig. 3:**
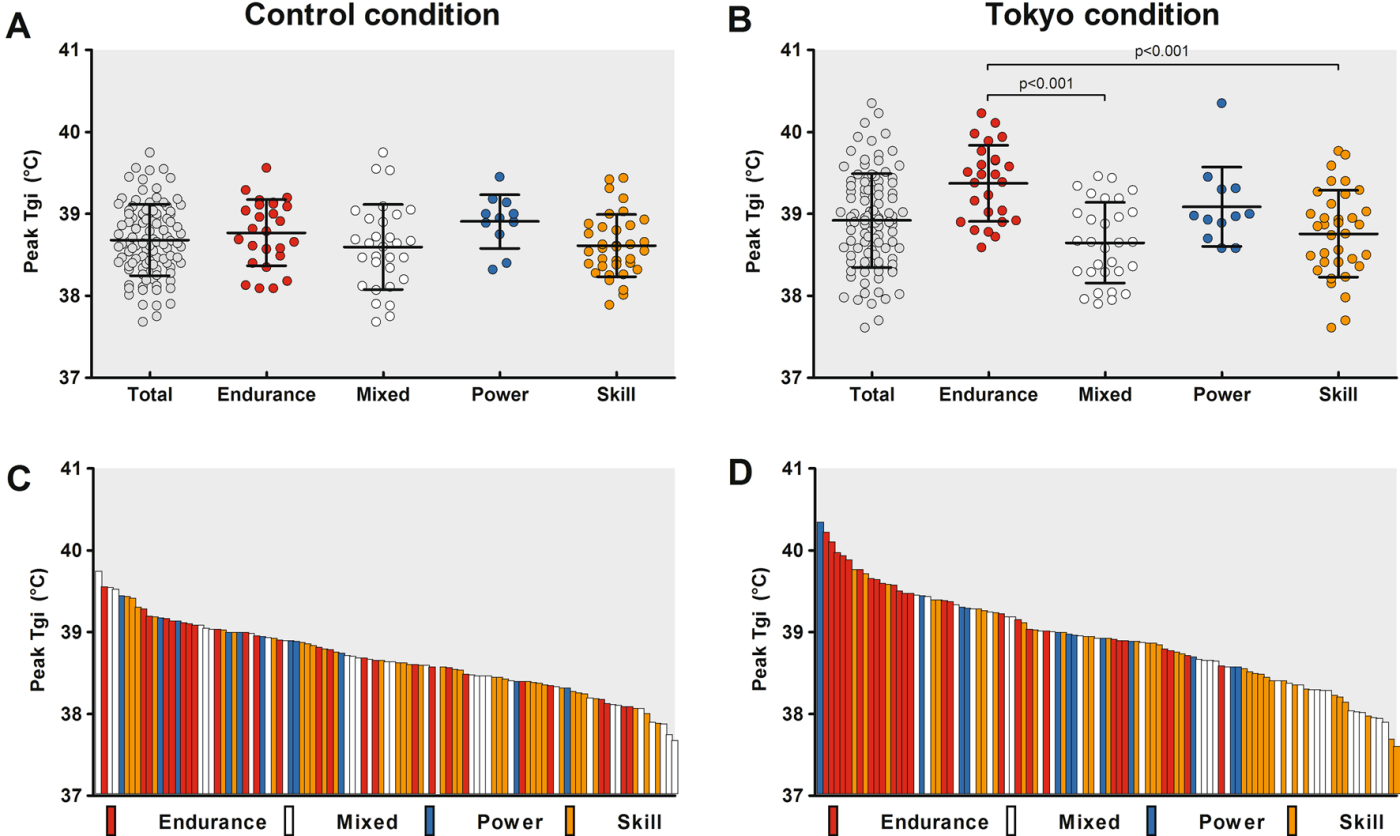
Group data (panels **A** + **B**) and individual data (panels **C** + **D**) of peak gastrointestinal temperature (*T*_gi_) achieved during the control (panels **A** + **C**) and Tokyo (panel **B** + **D**) conditions. Data in the upper panels are presented as mean ± SD. Endurance athletes demonstrated a significantly higher peak *T*_gi_ in the Tokyo condition compared to mixed and skill athletes. Each bar of panels C and D represent data from an individual athlete, whereas the largest interindividual variability can be observed in the Tokyo condition

**Fig. 4:**
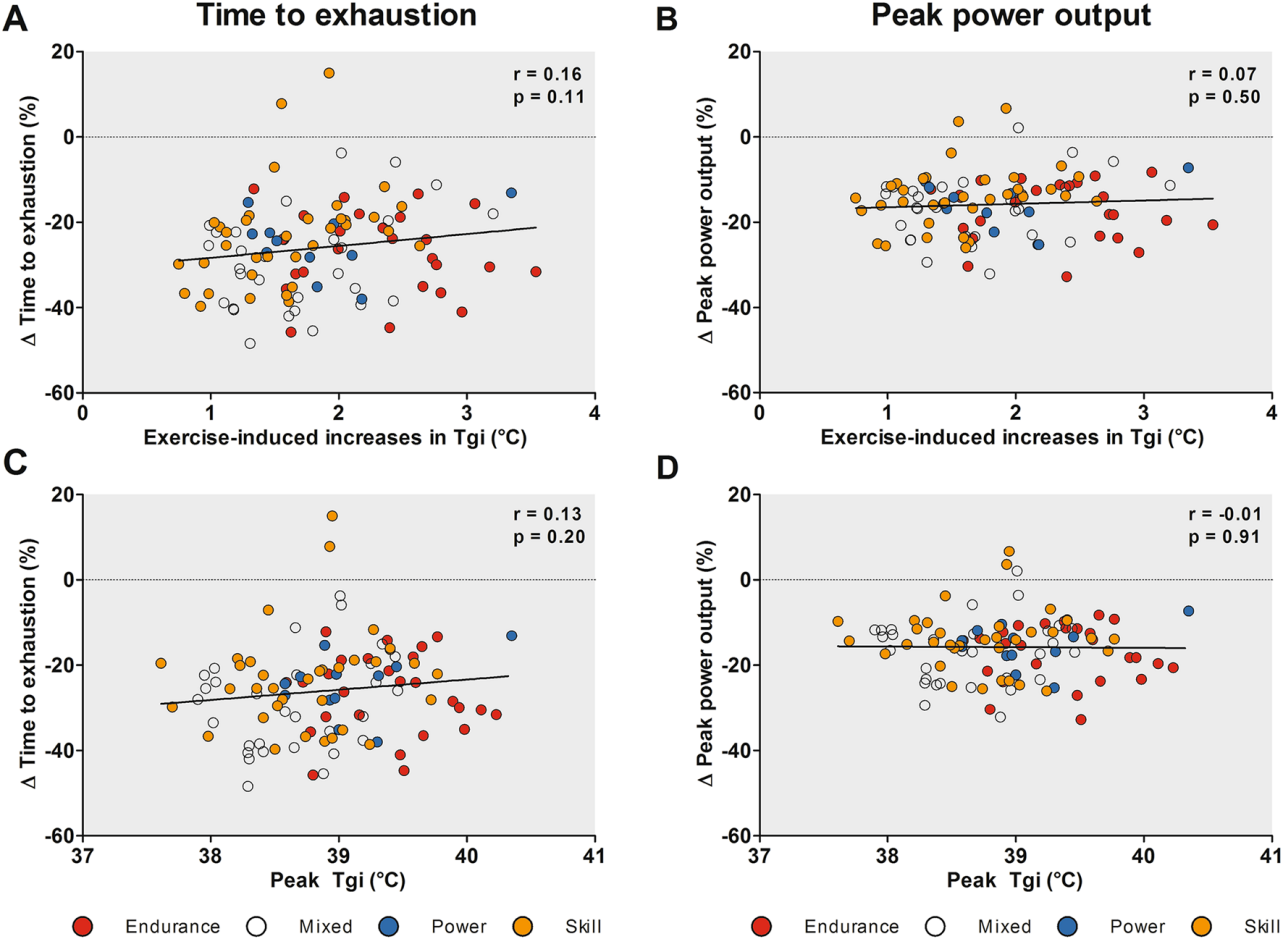
Correlations between percentual changes in time to exhaustion (panels **A** + **C**) and peak power output (panels **B** + **D**) and the exercise-induced *T*_gi_ increase (panels **A** + **B**) and peak *T*_gi_ (panels **C** + **D**) in the Tokyo condition. Neither peak *T*_gi_ nor exercise-induced increase in *T*_gi_ were associated with the changes in time to exhaustion or peak power output between the Tokyo and control conditions

Resting *T*_gi_ was comparable across conditions (*p* = 0.30, Table 2), but larger exercise-induced *T*_gi_ increases, higher *T*_gi_ rates and higher peak *T*_gi_ values were found in the Tokyo versus control condition (all *p* values < 0.001, Table 2). The distinct pattern in peak *T*_gi_ responses was even more evident after standardization for exercise duration (Fig. 2A). Larger interindividual variations in exercise-induced increases in *T*_gi_ (range 0.7–3.5 °C vs. 0.7–3.0 °C) and peak *T*_gi_ (range 37.6–40.4 °C vs. 37.7–39.7 °C) were found across athletes in the Tokyo condition compared to the control condition (Fig. 3D). Peak *T*_gi_ values were moderately correlated across both conditions (Pearson’s *r* = 0.59, *p* < 0.001). There were no associations between peak *T*_gi_ or exercise-induced increase in *T*_gi_ and the relative changes in TTE or PPO (Fig. 4). Furthermore, endurance-trained athletes demonstrated greater exercise-induced increases in *T*_gi_ (Table 3) and reached higher peak *T*_gi_ values (Fig. 3B) compared to mixed- and skill-trained athletes in the Tokyo condition. Moreover, endurance-trained athletes demonstrated a higher *T*_gi_ rate in the Tokyo condition compared to skill-trained athletes (Table 3). Additional comparisons of thermoregulatory responses across sport disciplines are presented in Table 3.

Resting *T*_sk_, exercise-induced increase in *T*_sk_, and peak *T*_sk_ were higher in the Tokyo versus the control condition (all *p* values < 0.001, Table 2, Fig. 2B). Exercise-induced increases in *T*_sk_, and peak *T*_sk_ were higher in endurance-trained athletes compared to mixed- and skill-trained athletes across both conditions (Table 3).

### Heart Rate, Whole Body Sweat Rate and Subjective Outcomes

Resting HR was higher in the Tokyo versus control condition (*p* < 0.001, Table 2), increased during both conditions (Fig. 2C), whereas greater exercise-induced increases and higher peak values were found in the Tokyo versus control condition (both *p* < 0.001, Table 2). Sport discipline-specific differences in resting HR, exercise-induced increases in HR and peak HR were observed in both conditions (Table 3).

WBSR and dehydration levels were higher in the Tokyo versus control condition (both *p* < 0.001, Table 2). Endurance and mixed athletes demonstrated a higher WBSR compared to power athletes in the Tokyo condition (Table 3). Relative losses in total body weight were the highest in endurance trained athletes compared to mixed-, power- and skill-trained athletes in both conditions (Table 3).

Peak thermal sensation and peak thermal comfort scores deteriorated in the Tokyo compared to the control condition (all *p*s < 0.001, Table 2). Maximum RPE did not differ across conditions (*p* = 0.11). Higher peak thermal comfort scores were observed in power-trained athletes compared to endurance- and mixed-trained athletes (Table 3).

## Discussion

The current study provides a unique insight into the changes in exercise performance and thermoregulatory responses among a large and heterogeneous cohort of elite athletes exercising in hot and humid environmental conditions as compared to control conditions. As expected, larger exercise-induced increases in *T*_gi_ were observed in the Tokyo versus control condition. The magnitude of these thermoregulatory responses was more prominent in endurance-trained athletes compared to mixed- and skill-trained athletes, whereas thermal perception was more affected in power-trained athletes. More importantly, a dramatic loss in exercise performance was found (− 26 ± 11%), independent of sports discipline. Also, large interindividual variations were present for peak *T*_gi_ and performance loss, whereas no association was found between these outcome measures. Findings from this study indicate that the Tokyo environmental conditions have a significant impact on the thermoregulatory responses and performance capacity of Dutch elite athletes. The magnitude of physiological responses to heat is highly variable across individuals, underlining the need for individualized heat mitigation strategies to optimize exercise performance and to minimize the potential risk of heat illness in preparation for the Tokyo Olympics.

### Exercise Performance

TTE declined by 26 ± 11% in the Tokyo condition, with 25% of the athletes demonstrating a performance loss > 35%. The magnitude of this decrement is lower compared to previous lab-based studies in low-to-moderate-trained individuals (range − 29% to − 48%) [8, 31–33]. Study-specific differences in exercise protocol, intensity (absolute workload), duration and environmental conditions may contribute to the observed variability of heat-induced performance loss. Alternatively, regular exposure to high-volume and/or high-intensity exercise training is known to induce partial heat acclimatization [34–36], which could explain the attenuated heat-induced performance loss in elite athletes compared to low-to-moderate-trained individuals who were primarily tested in previous studies. Two of our participants even demonstrated an improved exercise capacity in the heat. A case-by-case review revealed no abnormalities in physiological responses or exercise performance parameters, highlighting that the continuum of changes in exercise performance in the heat ranges from large decrements to small improvements. Taken together, exercise in the heat induces large reductions in performance for the majority of elite athletes.

### Thermoregulatory Responses

A larger increase in *T*_gi_ was observed in the Tokyo versus control condition. This finding aligns with our hypothesis and observations from previous studies [8, 37, 38], as the hot and humid environmental conditions narrow the heat transfer gradients between the skin surface and the ambient environment, attenuating the possibility of dissipating heat generated by skeletal muscles [39]. The high humidity and the lack of wind speed further limit the possibility of evaporation, resulting in a significantly lower heat-loss potential [40]. The limited heat-loss potential in the Tokyo condition resulted in a distinct pattern in time-dependent changes of *T*_gi_ across conditions (Fig. 2A). Increases in *T*_gi_ level-off after 20 min in the control condition, whereas a continuous rise until exercise cessation is observed in the Tokyo condition. These findings may indicate uncompensatable heat stress in the Tokyo condition [41], which might result in severe hyperthermia and an increased risk for heat illness if exercise is performed at higher absolute workloads and/or continued for a longer time.

### Subjective Measures

Power-trained athletes reported higher rest and peak thermal comfort scores compared to endurance- and mixed-trained athletes. This is an interesting observation as thermal perception can affect exercise performance independent of core temperature changes [42, 43]. Skin temperature is the primary driver of thermal comfort during exercise in the heat [29, 44], but peak exercise *T*_sk_ was lower in power versus endurance athletes and comparable between power- and mixed-trained athletes. On the other hand, the difference in peak exercise *T*_sk_ between the control and Tokyo conditions was the largest for power-trained athletes (Table 3), but this value did not significantly differ from other athlete groups. Alternatively, body composition is known to impact thermal comfort scores [45]. Power athletes had a higher body mass index (BMI; 25.4 ± 1.3 kg/m^2^) compared to endurance- (20.9 ± 1.4 kg/m^2^) and mixed-trained athletes (20.9 ± 1.4 kg/m^2^, *p* < 0.001), which may contribute to the difference in thermal perception scores [45]. Nevertheless, we must also acknowledge that performance loss was not different for power athletes, so their worse thermal comfort score did not result in an additional attenuation of exercise performance in the heat.

### Interindividual Variations

We observed large interindividual variations in thermoregulatory responses with a peak *T*_gi_ ranging from 37.6 to 40.4 °C in the Tokyo condition. Although the level of hyperthermia was moderate for the whole cohort (peak *T*_gi_ 38.9 ± 0.6 °C) and aligned with previous observations in literature [8, 31–33], substantial differences were found across sport disciplines. Endurance-trained athletes showed larger increases (2.3 ± 0.6 °C) and higher peak values of *T*_gi_ (39.4 ± 0.5 °C) compared to mixed- (1.7 ± 0.6 °C and 38.6 ± 0.5 °C) and skill- (1.6 ± 0.5 °C and 38.8 ± 0.5 °C) trained athletes. The difference in absolute peak workload (and associated metabolic heat production) per unit mass between endurance (3.7 ± 0.5 W/kg) mixed- (2.5 ± 0.3 W/kg) and skill-trained athletes (1.9 ± 0.4 W/kg) is likely responsible for this finding [46] as exercise duration was comparable across groups (Table 1). Therefore, endurance-trained athletes were in an uncompensatable state for a longer period compared to other groups, given the higher workload per unit mass. In contrast to the thermoregulatory responses, no differences in performance decrements were observed across sport disciplines. This observation suggests that endurance-trained athletes may have a better heat tolerance compared to other sport disciplines. The lack of an overall association between peak *T*_gi_ and performance outcomes (Fig. 4) further substantiates this hypothesis as within-subject variations in *T*_gi_ are known to impact performance capacity [47], but between-subject variations may reflect differences in heat tolerance instead. Taken together, the magnitude of physiological responses is highly variable across elite athletes in simulated Tokyo 2020 conditions, emphasizing the importance of testing athletes to determine their individual needs for specific heat-mitigation measures.

### Individualized Countermeasures

The large interindividual variations, the differences observed across sport disciplines, and the absence of a significant association between *T*_gi_ and performance indicators emphasize that it is impossible to offer a ‘one-size-fits-all’ heat-mitigation strategy to elite athletes. Participants in the present study were non-acclimatized, indicating that the physiological responses represent a worst-case scenario, but also highlight a large window of opportunity to improve thermoregulation and exercise performance in the heat. Heat acclimatization is the most important countermeasure one can adopt [48], and must be implemented in the training plan of an athlete [15, 18]. Cooling interventions [16, 17, 49] and hydration strategies [50] are other effective measures to attenuate the deleterious effects of exercise in the heat. Each heat-mitigation strategy should be individualized based on the athlete’s thermophysiological responses and performance losses during exercise in the heat, and must be practiced during training and competition prior to the Olympic Games.

### Strengths and Limitations

Our study used a well-controlled maximal exercise protocol and included a unique and large group of 105 elite athletes, in order to provide a comprehensive insight into the effects of the Tokyo environmental conditions on thermoregulatory responses and exercise performance. However, some limitations should be taken into account. First, a possible weakness of our study is the lack of a randomized study design, as the first exercise test was always conducted in the control condition to obtain the heart rate-based personalized exercise protocol (i.e. changes in workload over time) for both conditions. We preferred this approach over a time-trial performance protocol, as the latter may induce additional variability in performance and pacing levels in athletes inexperienced in such types of exercise (e.g., field hockey, sailing, beach volleyball) due to learning effects [51]. We also considered adding a familiarization session to our protocol, allowing randomization of the two other sessions. However, representatives of TeamNL and sports federations advised against such an approach due to the busy training schedules of elite athletes and the impracticability of exposing them to multiple exercise tests within a short period. Therefore, we deliberately chose the current approach that allows us to use personalized protocols, and obtain thermoregulatory responses and performance outcomes by two study visits only. Second, athletes were not allowed to drink during the exercise protocol to avoid interference with the measurements of the ingestible temperature capsule [24], but this may also have exaggerated the effect of heat on thermoregulation and performance due to accelerated dehydration [52]. Third, the exercise protocol does not reflect the nature of sport-specific activities and related (behavioural) thermoregulatory responses, limiting the external validity and potentially a direct translation to field settings. However, outcomes of our standardized exercise test have a high internal validity and may aid coaches in specifically collecting field data in athletes with abnormal responses, such as a large performance loss or high peak T_gi_, which aligns with the goal of the Thermo Tokyo study.

## Conclusion

Exercise performance was severely affected among non-acclimatized elite athletes exercising in a hot and humid environment as evidenced by a 26 ± 11% lower time to exhaustion. We also observed larger increases of core temperature and skin temperature in the Tokyo versus the control condition, with aggravated responses in endurance athletes (greater exercise-induced increases in *T*_gi_ and peak *T*_gi_) and power athletes (deteriorated thermal perception). The magnitude of performance loss and thermoregulatory responses were highly variable across individuals, whereas no association was found between these outcome measures. These findings emphasize the importance of extensive testing of elite athletes to determine their individual needs for heat mitigation strategies (i.e. heat acclimatization, cooling interventions, hydration plan). Such an approach will likely contribute to safe and maximal exercise performance during the challenging environmental conditions of the Tokyo 2020 Olympic Games and other future competitions in challenging hot and humid environments.

## Supplementary Information

Below is the link to the electronic supplementary material.

Supplementary file1 (PDF 86 kb)

Supplementary file2 (PDF 178 kb)

## Declarations

### Availability of data and material

Data are available upon reasonable request.

### Twitter

@YannickdeKorte, @BongersCoen, @ThijsEijsvogels.
