# Supplementary material for: Exercise Performance and Thermoregulatory Responses of Elite Athletes Exercising in the Heat: Outcomes of the Thermo Tokyo Study
Source: Sports Med. 2021 Aug 15;51(11):2423–36. doi: 10.1007/s40279-021-01530-w (PMC8514392; doi:10.1007/s40279-021-01530-w)
Supplement: Supplementary file 1 — Supplementary file1 (PDF 86 kb) [file 40279_2021_1530_MOESM1_ESM.pdf]

Exercise performance and thermoregulatory responses of elite athletes exercising in the heat: outcomes of the Thermo Tokyo study

Running heading: *Exercise performance and thermoregulatory responses of elite athletes exercising in the heat*

Johannus Q. de Korte, M.Sc.[1]

Coen C.W.G. Bongers, PhD.[1]

Maria T.E. Hopman, M.D. Ph.D.[1]

Thijs M.H. Eijsvogels, Ph.D.[1]

Affiliations:

[1] Radboud university medical center, Radboud Institute for Health Sciences, Department of Physiology, Nijmegen, The Netherlands.

Address for correspondence:

Dr. Thijs Eijsvogels, Department of Physiology (392), Radboud university medical center, P.O. Box 9101, 6500 HB Nijmegen, The Netherlands, Tel +31 24 36 13 674, Fax +31 24 36 16413,

E-mail: Thijs.Eijsvogels@radboudumc.nl

Supplementary Table 1: A fictive example of an individualized exercise protocol in the control and Tokyo condition. All athletes started at 100 W and after 3 min the initial workload was gradually adjusted (on the minute marks) to reach 70% of the athlete's maximal HR. When a stable target HR was reached (i.e. 70% HRmax), the workload was kept equal for the remaining minutes of the 20 min warm-up. At the 20 min mark, the incremental phase started and the workload (in W) was increased every 3 minutes by 5% of the workload corresponding to 70% HRmax until volitional exhaustion. The personalised exercise protocol (i.e. changes in workload over time) obtained during the control condition was subsequently applied to the second exercise test in simulated Tokyo conditions. The maximal heart rate in this example is 200 bpm.

		Control condition		Tokyo condition		
Phase	Minute	Heart rate (bpm)	Workload (W)	Heart rate (bpm)	Workload (W)	
20 min warm - up phase	0	60	100	68	100	
	1	65	100	73	100	
	2	70	100	78	100	
	3	72	110	81	110	
	4	85	120	95	120	
	5	95	130	112	130	
	6	110	140	121	140	
	7	123	150	135	150	
	8	127	160	139	160	
	9	129	170	148	170	
	10	136	180	151	180	
	11	139	190	154	190	
	12	140	200	158	200	**Target HR reached**
	13	141	200	161	200	
	14	142	200	160	200	
	15	141	200	162	200	
	16	140	200	163	200	
	17	142	200	164	200	
	18	143	200	164	200	
	19	141	200	163	200	
Incremental phase	20	141	210	165	210	**+ 10 W / 3 min**
	23	142	220	168	220	
	26	143	230	172	230	
	29	148	240	176	240	
	32	151	250	184	250	
	35	156	260	191	260	
	38	163	270	197	270	**Volitional exhaustion**
	41	171	280			
	44	178	290			
	47	182	300			
	50	187	310			
	53	190	320			
	56	193	330			
	59	196	340			**Volitional exhaustion**
Cool-down	1	165	75	174	75	
	2	154	75	159	75	
	3	138	75	143	75	
