# Supplementary material for: Exercise Performance and Thermoregulatory Responses of Elite Athletes Exercising in the Heat: Outcomes of the Thermo Tokyo Study
Source: Sports Med. 2021 Aug 15;51(11):2423–36. doi: 10.1007/s40279-021-01530-w (PMC8514392; doi:10.1007/s40279-021-01530-w)
Supplement: Supplementary file 2 — Supplementary file2 (PDF 178 kb) [file 40279_2021_1530_MOESM2_ESM.pdf]

Exercise performance and thermoregulatory responses of elite athletes exercising in the heat: outcomes of the Thermo Tokyo study

Running heading: *Exercise performance and thermoregulatory responses of elite athletes exercising in the heat*

Johannus Q. de Korte, M.Sc.[1]

Coen C.W.G. Bongers, PhD.[1]

Maria T.E. Hopman, M.D. Ph.D.[1]

Thijs M.H. Eijsvogels, Ph.D.[1]

Affiliations:

[1] Radboud university medical center, Radboud Institute for Health Sciences, Department of Physiology, Nijmegen, The Netherlands.

Address for correspondence:

Dr. Thijs Eijsvogels, Department of Physiology (392), Radboud university medical center, P.O. Box 9101, 6500 HB Nijmegen, The Netherlands, Tel +31 24 36 13 674, Fax +31 24 36 16413,

E-mail: Thijs.Eijsvogels@radboudumc.nl

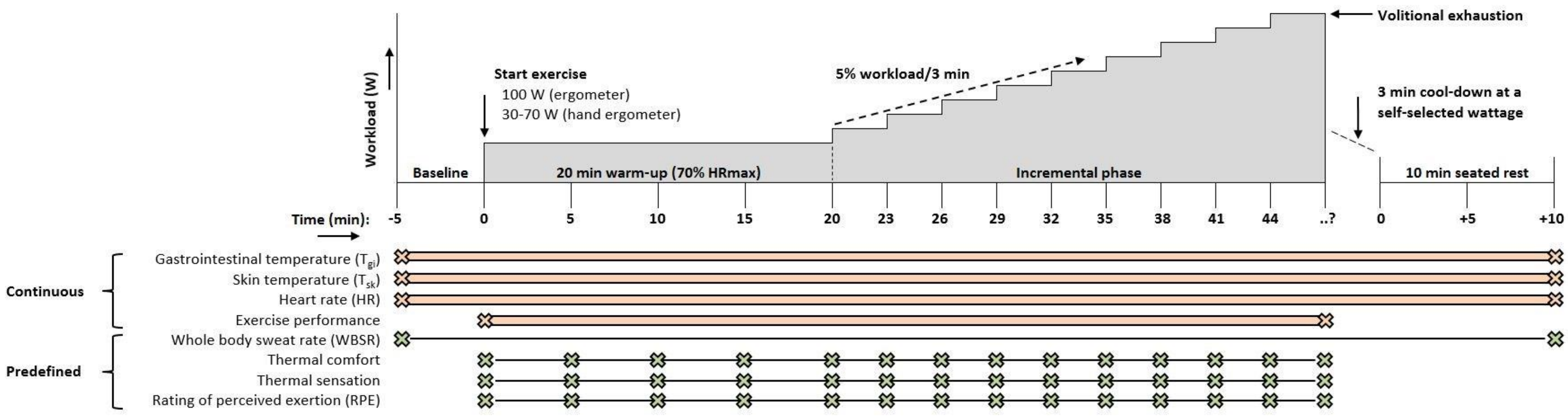

Supplementary Figure 1: Overview of the personalised exercise test protocol**.** After 5 min seated rest (Baseline), athletes started to exercise at 100W. After 3 min, the initial workload was gradually adjusted to reach 70% of the athlete's maximal heart rate. After 20 min, workload gradually increased every 3 min with 5% until volitional exhaustion. After exercise cessation, participants had a 3 min cool-down at a self-selected wattage followed by 10 min seated rest. Gastrointestinal temperature (T_{gi}), skin temperature (T_{sk}), heart rate (HR), and exercise performance were examined continuously throughout the protocol. Participant's body mass was measured at baseline and directly after finishing the experimental protocol to determine whole body sweat rate (WBSR). Perceptual outcomes were scored at baseline, every 5 min during the warm-up phase and every 3 min during the incremental phase. The personalised exercise protocol (i.e. changes in workload over time) obtained during the thermoneutral condition ($T_{ambient}$ 15.9±1.2°C, RH 55±6%) and was subsequently applied to the second exercise test in simulated Tokyo condition ($T_{ambient}$ 31.6°C±1.0, RH 74±5%).
